# Activation of Cre Recombinase Alone Can Induce Complete Tumor Regression

**DOI:** 10.1371/journal.pone.0107589

**Published:** 2014-09-10

**Authors:** Yulin Li, Peter S. Choi, Stephanie C. Casey, Dean W. Felsher

**Affiliations:** Department of Medicine, Division of Oncology, School of Medicine, Stanford University, Stanford, California, United States of America; Sanford Burnham Medical Research Institute, United States of America

## Abstract

The Cre/loxP system is a powerful tool for generating conditional genomic recombination and is often used to examine the mechanistic role of specific genes in tumorigenesis. However, Cre toxicity due to its non-specific endonuclease activity has been a concern. Here, we report that tamoxifen-mediated Cre activation *in vivo* induced the regression of primary lymphomas in *p53−/−* mice. Our findings illustrate that Cre activation alone can induce the regression of established tumors.

## Introduction

The Cre/loxP system has been widely used to induce tissue- and developmental stage- specific genomic recombination [1], [2]. Cre/loxP is a particularly tractable strategy to examine the role of gene activation or inactivation in the initiation and maintenance of tumorigenesis. However, Cre activation can also induce non-specific genomic recombination. In some cases, Cre activation has been shown to induce significant cellular toxicity associated with marked cell death in cell culture [3]–[5]. This toxicity is not seen in Cre mutants that do not have endonuclease activities [3].

Similarly, Cre activation *in vivo* in mouse models can induce toxicity in normal cellular lineages. For example, in mouse lines with Cre expressed in neuronal progenitors, defects have been observed in brain development [6]. Myocardial-specific Cre activation with tamoxifen in the α*MHC-MERCreMER* mice induces cardiac fibrosis and heart failure [7], [8]. Furthermore, systemic Cre activation with tamoxifen in the *Rosa26-CreER^T2^* mice results in thymic atrophy and severe hematological toxicity [9].

The Cre/loxP system has been used to interrogate the role of specific genes in tumorigenesis [2]. Although most studies have presumed that Cre does not have effects on tumorigenesis, one report described that Cre expression blocked tumor formation in a mouse model of lymphoma transplantation [10]. Here we report that Cre activation resulted in the regression of primary lymphoma induced by p53 deficiency. Our results have implications for the use of the Cre/loxP system for tumorigenesis studies.

## Methods

### Lymphoma model

All animal work was approved by the Stanford IACUC committee (protocol number 14045) and follows AAALAC guidelines. The p53 deficient mice (*p53−/−*) were used as the spontaneous lymphoma model. The *p53−/−* mice were maintained in the FVB/N background and the majority of them developed malignant lymphoma within 6 months of age [11]. The *UBC-Cre-ER^T2^* mice carrying the transgenic *Cre-ER^T2^* controlled by the Ubiquitin C (*UBC*) promoter were maintained in the 129S6 background [12]. The *p53−/−* mice were crossed with the *UBC-Cre-ER^T2^* mice to derive the *UBC-Cre-ER^T2^; p53−/−* mice.

### Conditional Cre activation

For *in vivo* activation of Cre, the mice were gavaged with tamoxifen (200 mg/kg, once daily, with one day off after 4 consecutive doses) for 9 days. For *in vitro* activation of Cre, the *p53−/−* lymphoma cell lines carrying either *MSCV* empty vector or *MSCV-Cre-ER^T2^* were treated with 1 micromolar of 4-hydroxytamoxifen (Sigma, H7904) for 48 hours.

### MRI imaging

Lymphoma development in the mice was monitored using magnetic resonance imaging (MRI). Once the lymphoma was established, mice were treated with tamoxifen. The tumors were imaged before treatment and 12–16 days after the start of treatment with a 7T MRI system (T2-weighed fast spin echo) at Stanford small animal imaging facility. The MRI image stacks were analyzed with the Osirix image application to derive the tumor volumes.

### Flow cytometric analysis of apoptosis

For detection of apoptosis, the lymphoma cells were stained with Annexin V and 7-AAD and analyzed with a FACSCalibur (BD Biosciences). The apoptotic cell populations were visualized with FlowJo (Treestar).

### Immunohistochemical analysis of apoptosis

For detection of apoptosis *in vivo*, the thymic lymphomas from mice treated with tamoxifen were fixed, paraffin-embedded and sectioned. Tissue slides were stained with a cleaved-Caspase 3 antibody (Cell Signaling #9661) following manufacturer’s instructions. The slides were developed with DAB (Vector Laboratories) and also counterstained with hematoxylin.

## Results and Discussion

**Figure 1:**
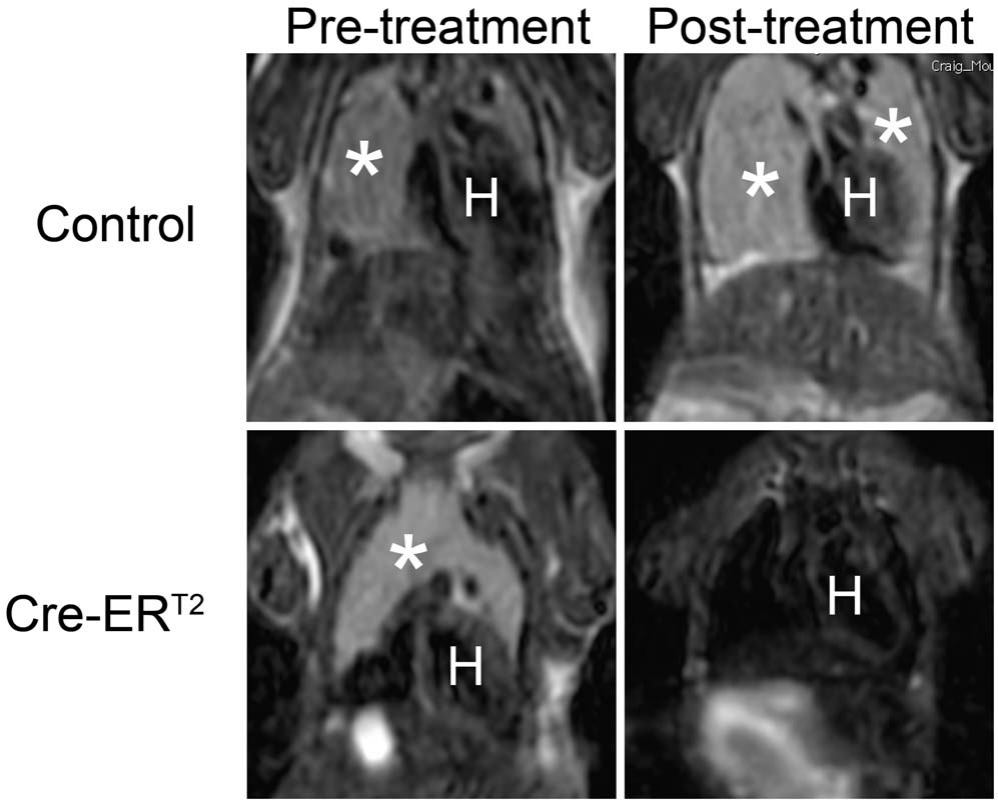
Changes in tumor volume upon tamoxifen treatment in control *p53−/−* and *UBC-Cre-ER^T2^; p53−/−* mice as shown by MRI imaging. The coronal sections of the thymic lymphoma were shown with tumors labeled with white asterisks. The letter H denotes the location of heart. Post-treatment scans were performed 14 days after starting tamoxifen treatment.

**Figure 2:**
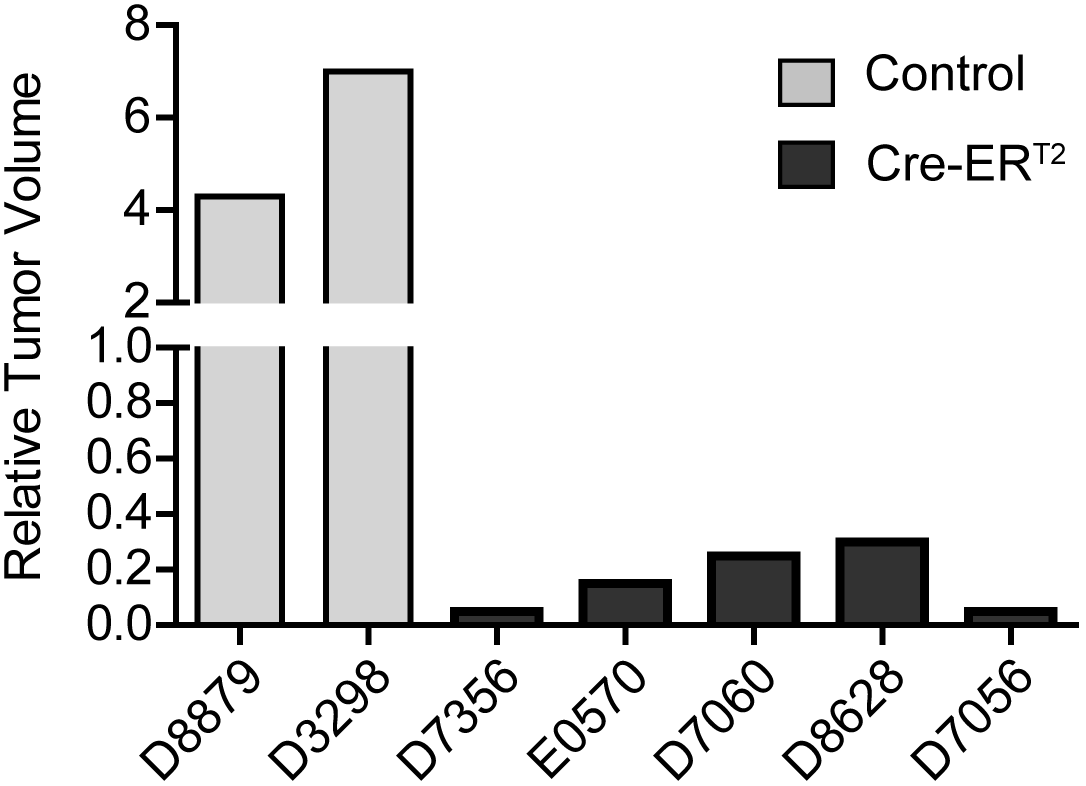
Summary of tumor volume changes upon tamoxifen treatment. Relative tumor volume was calculated by dividing post-treatment tumor volume by pre-treatment tumor volume. Unpaired t test, p<0.001. The code number of each mouse is labeled on the x-axis.

The p53 deficient mice (*p53−/−*) were used as the spontaneous lymphoma model. As previously reported, the *p53−/−* mice developed malignant lymphoma within 6 months of age [11]. The *UBC-Cre-ER^T2^* was introduced into the *p53−/−* background by crossing. Upon Cre activation with tamoxifen treatment, the temporal regression of *UBC-Cre-ER^T2^; p53−/−* lymphoma was observed in multiple independent tumors as measured by MRI imaging (Figure 1). Tumor volume quantification using MRI image stacks showed that the size of the *UBC-Cre-ER^T2^; p53−/−* lymphoma was reduced to 0%–30% of the pretreatment level (Figure 2). The regression of the tumors was also visually confirmed by postmortem dissection. In contrast, the volume of the *p53−/−* lymphoma without *UBC-Cre-ER^T2^* transgene increased 4–6 fold despite tamoxifen treatment (Figure 2).

**Figure 3:**
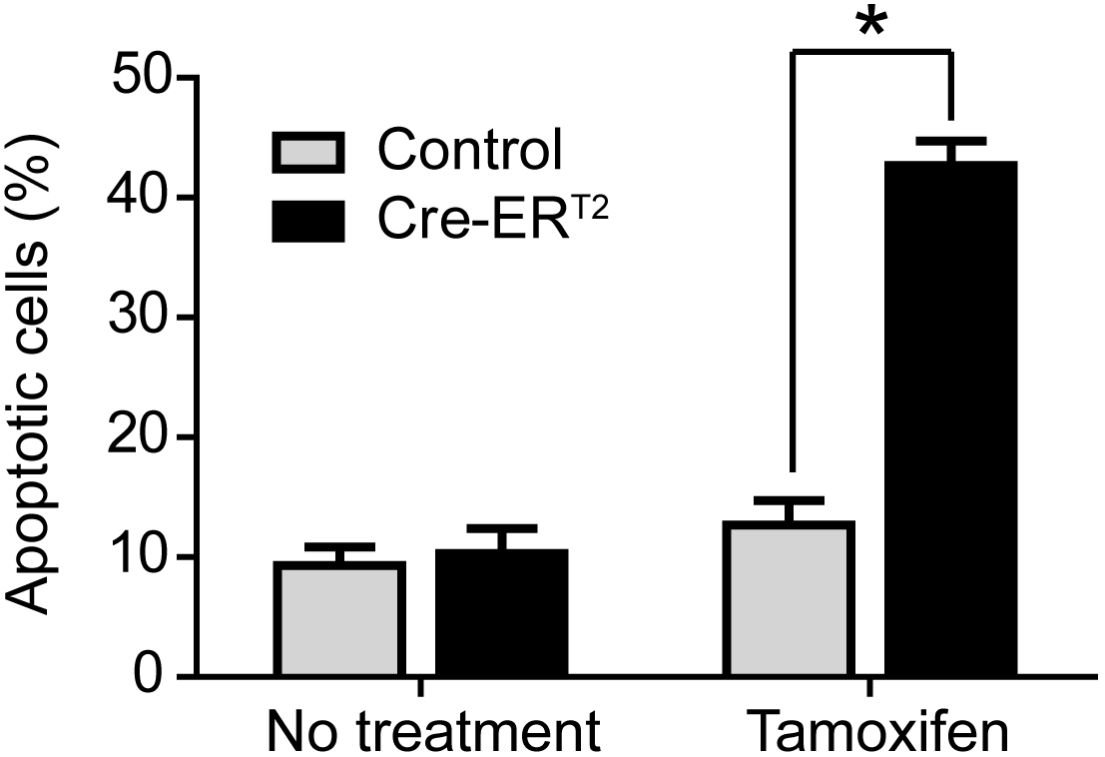
*In vitro* Cre-ER^T2^ activation with tamoxifen in *p53−/−* lymphoma cells results in apoptosis. Cells were treated in triplicates with 1 micromolar 4-hydroxytamoxifen for 48 hours and analyzed with flow cytometry after Annexin V/7-AAD staining. * Paired t test, p<0.005.

**Figure 4:**
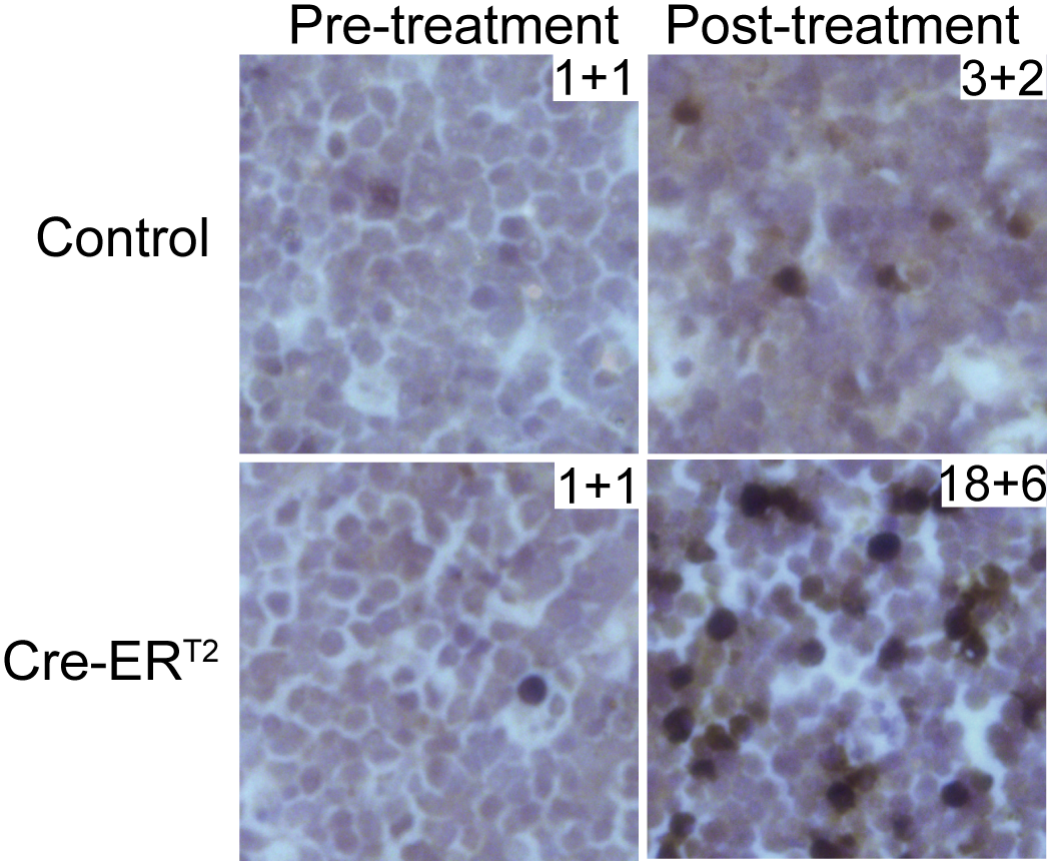
*In vivo* Cre-ER^T2^ activation with tamoxifen results in apoptosis in primary *p53−/−* lymphoma. Mice with thymic lymphomas were treated with tamoxifen. Tumor sections were stained with a cleaved-Caspase 3 antibody. Apoptotic cells were stained brown color. The numbers in the top right corners represent the percentage of apoptotic cells. Data are presented as mean + standard deviation. Paired t test p<0.01, for Cre-ER^T2^ post-treatment versus pre-treatment.

To investigate the mechanism of lymphoma regression, we tested whether Cre activation could induce apoptosis in a *p53−/−* lymphoma cell line derived from the mouse model. *Cre-ER^T2^* was overexpressed in the *p53−/−* lymphoma cell line with the retroviral Murine Stem Cell Virus (*MSCV-Cre-ER^T2^*). Upon Cre activation in cell culture with one micromolar of 4-hydroxytamoxifen, there was a four-fold increase of apoptotic cells (11% to 42%) as shown by flow cytometric analysis after Annexin V/7-AAD staining. In contrast, the control *p53−/−* lymphoma cell line carrying an empty *MSCV* vector did not show significant changes in apoptosis rate (Figure 3). To further test whether Cre activation could induce apoptosis *in vivo*, we stained the *p53−/−* thymic lymphomas using a cleaved-Caspase 3 antibody. Tamoxifen treatment induced a significant increase in cleaved-Caspase 3 staining (1% to 18%) in the *p53−/−*; *UBC-Cre-ER^T2^* tumors but only moderate changes in the control *p53−/−* tumors (Figure 4). Hence, Cre activation induced significant cell death which can contribute to the *in vivo* tumor regression in the *p53−/−* mouse model.

Our results are the first to demonstrate that Cre activation alone can induce the regression of established primary tumors *in vivo*. We found that Cre activation can induce marked apoptosis in *p53−/−* lymphoma. Our results are consistent with prior reports that Cre activation can result in toxicity in normal tissues [6]–[9]. The most likely explanation for our finding is that Cre activation induces genomic rearrangement associated with the cryptic loxP sites within the mouse genome [9]. We recognize that the effects of Cre are likely dose- and duration-dependent.

Our findings reinforced the previous notion that, for studies with the Cre/loxP system, an experimental control using mice with Cre expression but without the loxP sites should always be included for the possibility that Cre activation can markedly perturb the initiation and progression of tumorigenesis and even induce the regression of established tumors [10].
